# Before narrative: episodic reading and representations of chronic pain

**DOI:** 10.1136/medhum-2017-011223

**Published:** 2018-01-05

**Authors:** Sara Wasson

**Keywords:** literary theory, literature and medicine, narrative medicine, pain management, patient narratives

## Abstract

This article suggests that some illness experience may require a reading practice less concerned with narrative coherence or self-authorship, and more interested in the value of textual fragments, episodes and moments considered outside a narrative framework. Chronic pain can pose multiple challenges to the narrative orientations celebrated in both ‘survivorship’ discourse and classic medical humanities scholarship. In its recalcitrance to cure, its often mysterious aetiology and its complex blend of somatic, interpersonal and affective elements, representations of chronic pain can require a richer vocabulary of temporality. I draw on contemporary affect theory to augment the available critical vocabulary for the textual representation of protagonists’ temporal orientation within illness experience, identifying a language for the emergent present that resists a narrative form. Beyond identifying narrative ‘incoherence’, affect discourse gives a way to recognise the strained, equivocal *labour* of incoherence, of inhabiting a cryptic present moment. Affect theory’s attention to the emergent present may give a way to read incoherent ‘chaos’ outside from a narrative framework, not only as a dark, formless stage in a personal story. To expand our vocabulary for this position, I offer a term for a particular affective experience of the present amid repeated marginalisation: the temporality of thwarted connection. I illustrate how these concepts can enable an alternative reading stance by offering a brief analysis of Lous Heshusius’s hybrid autobiography and academic study, *Chronic Pain from the Inside Out*.

Recent work in health humanities and critical medical humanities has sought to augment traditional approaches to narrativity.^1–10^ Building on such scholarship, this article seeks to broaden the way illness narratives of chronic pain are approached. First, I suggest that certain conventions of illness narrative can come to seem typical of experience in ways that may be detrimental to some living with chronic pain. I argue that some illness experience may require a parallel reading practice, reading less in search of narrative coherence or self-authorship and more interested in the value of textual fragments, episodes and moments considered outside a narrative framework. Second, I draw on contemporary affect theory to augment the available critical vocabulary for the textual representation of protagonists’ temporal orientation within illness experience, identifying a language for the emergent *present* that resists a narrative form. Third, I will illustrate how this approach can enable an alternative reading stance by offering a brief analysis of Lous Heshusius’s hybrid autobiography and academic study, *Chronic Pain from the Inside Out*.^11^ Reading this work ‘episodically’, that is, without a narrative arc as a critical focus, enables a critical approach less alert to the individual journey of a self-authoring patient and more attuned to the social context for chronic pain suffering and the complex temporality of the experience of structural marginalisation. This article is about reading differently, and in the process making space for experience which tends to be unhearable even within the capacious realms of medical and health humanities.

People living with chronic pain are particularly vulnerable to such erasure. A ‘silent epidemic’ and a ‘global public health priority’, chronic pain affects nearly 28 million people in the UK and 20% of the global adult population.^12–14^ Defined as pain that endures for more than 6 months, chronic pain can be as severe as acute pain, damages interpersonal relationships and increases suicide risk.^15–18^ Yet people enduring chronic pain are often oddly invisible, with healthcare practitioners, kin and employers failing to recognise the severity of their experience. Sufferers often endure a representational crisis, struggling to communicate their experience amid stigma and invisibility. In response, this article is part of a wider project seeking to expand the critical vocabulary around the analysis of chronic pain representation. Although I am exploring cases where pain involves suffering, that is not to say that ‘pain’ and ‘suffering’ are synonymous (and ‘disability’ of course does not necessarily involve either).^19^

## Narrative hierarchies and chronic pain

It is a widespread contention in narratology that a narrative is informed by a sense of the ending to which it moves.^20^ Peter Brooks notes that the telos ‘shapes a story and gives it a certain direction or intent of meaning’,^21^ and Lennard Davis coined the term ‘teleogenic’ to denote the way certain kinds of narration are informed by a sense of their close.^22^ As Sara Ahmed says, “Reading for narrative is reading for the direction of its point”.^23^ In this article, I argue that conventions of narrative telos are a key way that illness narrations achieve normative work, demonstrating modes of being ill that have moral authority within particular cultural milieux. To avoid marginalising vulnerable voices, it may be that we need a complementary critical stance less attentive to the narrative arc of a text—and as such less attentive to an individual’s ‘personal illness journey’.^24–27^ To put it another way, this is not just about multiplying alternative illness stories, but also making a space for story that does not fit the expected form of ‘story’ at all.^28^ I will briefly examine how certain dominant expectations of illness narrative create and legitimise particular protagonist temporal orientations, and then I will describe how chronic pain can challenge these representations as well as the critical postures they have tended to engender.

In the ‘survivor’ genre of illness memoir, people facing illness are urged to self-position as ‘fighters’, which denotes having a ‘positive’ attitude and complying with practitioner instruction.^29–31^ ‘Fighter’ rhetoric also recruits medical patients to biopolitical self-surveillance and lifestyle management.^32–34^ Internalising survivorship discourse requires proleptically positioning oneself within a particular narrative expectation, invested in an eventual imagined triumph. This moral stance is reinforced by a narrative arc of ‘restitution’ (to use Arthur Frank’s term), characterised by patient faith in medicine’s ability to restore health.^25^ This narrative expectation limits what stories can be recognised within popular contexts, and refusing this narrative prolepsis is framed as moral failure.^35–37^

By contrast, medical humanities scholarship has long challenged such triumphalist narration, as well as emphasising the need for patients’ stories to counter the detachment of biomedical discourse.^24–27^ Yet many of these classic texts, too, celebrate particular narrative typologies and certain ideal temporal orientations for the protagonist. A recurring theme is the necessity of being able to represent the experience of a narratively coherent self. Frank, for example, describes how during illness, one may lose:

the central resource that any storyteller depends on: a sense of temporality…. The illness story is wrecked because its present is not what the past was supposed to lead up to, and the future is scarcely thinkable…. The way out of narrative wreckage is telling stories… the self is being formed [anew] on what is told.^25^

In such a view, a particular coherence in one’s story and a particular temporal orientation are seen as indispensable for life to be bearable. Illness is often described in terms of a narrative crisis, being locked in a present without a sense of a coherent narrative of past and imagined future. Ruth Nadelhaft, for example, describes illness as ‘tak[ing] place in what seems an eternal present. Past health and future recovery vanish in the face of the endless formlessness and present tense of the experience of pain…. Literature offers form, structure, and the illusion of dimension to what was out of control and without limit’.^38^ Anne Hunsaker Hawkins suggests that illness narratives try to ‘restore to reality its lost coherence and… discover, or create, a meaning that can bind it together again’.^39^ Similarly, Rita Charon suggests that medical practitioners inhabit ‘vectored time’, a time within which they can act and understand their actions as causally related, while patients inhabit ‘a timeless enduring’, where past/present/future are blurred, causality is mysterious, and agency is compromised.^26^ Charon argues that ‘the narrating of the patient’s story is a therapeutically central act, because to find the words to contain the disorder and its attendant worries gives shape to and control over the chaos of the illness’.^40^ In these models, writing helps one endure suffering by restoring a narrative form for one’s experience—and as a corollary, restoring a proleptic orientation for oneself as an agent who can take steps towards an imagined future. This is not to say that anyone naively assumes they are guaranteed a particular outcome, and indeed many illness narrations end ambiguously.^41^ Rather, I am describing how the cultural dominance of particular narrative forms come to imply virtue within certain kinds of protagonist temporal orientation, specifically an expectation of beneficial transformation in time.

Within Frank’s formulations, for example, a ‘quest’ narrative protagonist ‘honour[s]’ illness ‘for the sense of purpose that can be discovered in it … opening oneself to be changed by its experience’; and within ‘broken narrative’, one achieves a modicum of coherence collaboratively, with others helping to formulate one’s story.^42^ Even when illness cannot be cured, the protagonist can have ‘intransitive hope’, remaining open to an unspecified but beneficial transformation to come.^25^ These responses are beautiful and powerful, and I in no way write to demean them. However, I do want to consider how when certain kinds of temporal orientation within a self-story are deemed indispensable to a bearable human life, we risk marginalising those who cannot or will not take that stance.

Chronic pain can pose multiple challenges to the narrative orientations celebrated in both ‘survivorship’ discourse and classic medical humanities scholarship. In its recalcitrance to cure, its often-mysterious aetiology and its complex blend of somatic, interpersonal and affective elements, representations of chronic pain can require a richer vocabulary of temporality. Chronic pain disrupts the assumptions of our ‘analgesic culture’ that expects pain to be diagnosable and remediable.^43^ As a result, people living with chronic pain occupy a liminal position, with the social peril that implies.^25 26 44–46^ David Morris describes the isolation often attendant on chronic pain, which ‘seems to build up walls of separation’, ‘surrounding [people] with silence’.^47^ Lara Birk writes in her autoethnography that severe pain ‘not only ruptured the coherence of my narrative, it precluded coherence as a narrative possibility'; in her case, she found ’the embodied narrative of the person in pain is unpredictable, unreliable, and seemingly unsuitable for communication’.^48^ People living with chronic pain describe how their experience is disbelieved when it does not accord with expected narrations.^48–51^ Norma Ware calls this process ‘delegitimation’, and the epistemic violence of such a process cannot be overstated.^51–53^ In many cases the physical suffering is described as less unbearable than the emotional suffering of being disbelieved: ‘no [experience] was as devastating… as the humiliation that resulted from having their subjective perceptions and sensations of illness either trivialized or dismissed as psychosomatic’.^51^ Many people living with chronic pain report that they are excluded, marginalised and disregarded, and a key part of this vulnerability stems from *narrative* transgression, the way they may not be able to adopt the proleptic subjectivity attendant on a particular teleogenic narrative. They may flout the narrative conventions to which illness experience should conform.

Scholars of class, feminism and postcoloniality have identified many ways in which a narratively coherent self is a cultural construction imbricated with privilege. Matti Hyvärinen *et al* warn:

The normative mission to find and value coherence marginalizes many narrative phenomena, omits non-fitting narrators, encourages scholars to read narratives obsessively from the perspective of coherence, and poses ethically questionable pressures upon narrators who have experienced severe political or other trauma.… [T]he imperative of coherence works to legitimise certain narratives while excluding or marginalising others from the narrative canon.^54^

Similarly, Laura Salisbury, drawing on Angela Woods, warns that ‘linear narratives that stress deep psychological continuities across time and expressive, confessional “I”… might privilege and render problematically universal modes of subjectivity and self-expression that are, in fact, culturally and historically contingent’.^6 9 10^ Building on such critique, I want to suggest that it may be beneficial to widen our temporal vocabulary around representations of chronic pain, both intra-textually in describing protagonists’ temporal orientations and extra-textually in describing the processes of critical activity.

Reading without seeking coherence is not new—it has always existed as a counterstrand in medical humanities. Leading proponents of a narrative sense of self readily acknowledge that formulating a coherent narrative may at times be impossible or ideologically problematic. Cheryl Mattingly, for example, suggests that sometimes ‘silences or half-told tales disclose more’ than a ‘well-told tale’.^55^ Frank describes representations of grief in which ‘the nature of the experience does not, cannot, and never will coalesce into a cohesive whole, as narrative traditions expect wholeness’,^56^ and warns that dominant narrative forms can contribute to moral insularity, since ‘people have often learned their stories too well, so that other stories sound wrong at best and less than human at worst’.^57^

Faced with these perils, one remedy is diversifying stories. However, arguments have also been made for complementing analysis of narrative typologies with a renewed focus on fragments, mixed-genre modes and non-narrative elements of textual representation such as metaphor, diction, syntax and intertextuality.^1 2 5–8 58–60^ Anne Whitehead suggests that ‘fragmentary or mixed-media narrative modes’ may be valuable in conveying ‘chaotic and contingent’ illness experience.^60^ Keir Waddington and Martin Willis remind us that ‘narratives need not be linear… nor need they offer logic, coherence, or temporal movement’.^8^ An important alternative for reading for coherence comes from social science studies of oral narration. Mattingly suggests that the term ‘narrative drama’ can capture the way we ‘follow a narrative suspensefully, always reminded of the fragility of events, for things might have turned out differently’.^61^ Mattingly describes ‘emergent narratives’, moments where a social interaction acquires a spontaneous narrative quality, the embodied experience (not language alone) performing a sudden shared frame of reference. In these moments, life is suddenly raised to a narrative form.^61^ Mattingly’s model is invaluable in recognising how narrative form can be a delicate, spontaneous thing co-created in interaction; this is very different from teleogenic written memoir. Yet Mattingly is still describing narratives, stories moving in particular directions. Judy Segal suggests that when we approach illness primarily in terms of narrative, ‘we may find certain conventional structures too readily available, and then the whole process of figuring out if there is something to be learned from illness experience is shortcircuited by the salience of those structures’.^24^ If reading for narrative is ‘reading for the direction of its point’^23^ and for the degree of narrative coherence or narrative drama, then I suggest that reading episodically is to read looking for a place to pause—to cease looking for the arc of the individual longitudinal journey and instead to consider how a particular scene constructs an emergent *present*. To clarify this approach, I will now draw on affect theory.

## Affect and temporality

Affect theory is concerned with the inseparable entanglement of the somatic, the social and (in some of its incarnations) the emotional.^62–67^ This scholarship seeks language to describe emergent, visceral, often inchoate forces: as Joel Burges and Amy Elias say, this scholarship ‘is the effort to understand the present as it plays out in somatic contexts’.^68^ Raymond Williams’s notion of ‘structures of feeling’ is helpful in considering the connections between visceral somatic experience, emotion, cognition and the social. In terms salient for the present discussion of chronic pain, Williams notes that structures of feeling are partially affective, in that they involve ‘a social experience which is still in *process*, often indeed not yet recognised as social but taken to be private, idiosyncratic, and even isolating’.^69^ Ahmed complements Williams’s ‘structures of feeling’ by noting that we should also think of affect in terms of ‘feelings of structure’, markers of the way social, economic and biopolitical ‘force and harm… [are] directed toward some bodies and not others’.^23^ In a similar vein, Ann Cvetkovich defines trauma not as an individual wound, but a social one: trauma is ‘a name for experiences of socially situated… violence’.^63^ Even experiences traditionally understood as wholly personal*—*such as physical pain*—*can be read as ‘feelings that open bodies to others’.^64^ As Marika Cifor says, ‘pain, like all emotion, is social’.^70^

Much affect theory examines the complex ways in which the present moment is shaped by suffering. Traditional trauma theory offers a powerful framework for understanding the ongoing impacts of catastrophic events,^71–73^ but several affect theorists find such models inadequate to capture the repeated and diffuse strain and injury mediated by structural inequity. Legacies of catastrophic events also reverberate in the present, each new tremor another intensity around which a particular mode of embodied suffering can accrete. Lauren Berlant speaks of ‘slow death’, the grinding down of vulnerable subjects that occurs not through dramatic events but through ‘structurally induced attrition… keyed to… membership in certain populations’.^73^ To express such processes, many affect theorists seek a language for the way any experience of the present moment is always incomplete, in process; in Berlant’s terms, the present is ‘a thing that is sensed and under constant revision’.^73^ Kathleen Stewart describes affect as symptom of the complex workings of biopolitical, economic and social pressures within the ‘weighted and reeling present’:

From the perspective of ordinary affects, things like narrative and identity become tentative though forceful compositions of disparate and moving elements: the watching and waiting for an event to unfold, the details of scenes, the strange or predictable progression in which one thing leads to another, the still life that gives pause, the resonance that lingers.^74^

Here, the present moment is read as a suspended and unpredictable site, suffused with lines of force social, political and personal. To put it another way, beyond identifying narrative ‘incoherence’, affect discourse gives a way to recognise the strained, equivocal *labour* of incoherence, of inhabiting a cryptic present moment. The emergent present is veined with lines of force somatic, emotional and social and may eventually find an as-yet-unknown meaning— but only retrospectively.^73^

Frank’s category of ‘chaos’ (anti)narrative is salient here, a narrative approximation of an anguished state in which the subject has lost any sense of agency and there is ‘absence of narrative order’, and no ‘discernable causality’.^26^ Such experience is by definition unrepresentable until after the fact. Frank’s discussions of these representations focus on their incoherence: ‘The lack of any coherent sequence is an initial reason why such stories are hard to hear; the teller is not understood as telling a ‘proper’ story. But more significantly, the teller of the chaos story is not heard to be living a ‘proper’ life, since in life as in story, one event is expected to lead to another’.^26^ While this category is invaluable, I suggest that a problem with it is the way that, like any typology, it may inadvertently limit critical response to ascribing the label. While a coherent ‘self’ may indeed be absent, there is still much to say about the way a text conveys the flux and flow of a tortured temporality. Affect theory’s attention to the emergent present may give a way to read incoherent ‘chaos’ outside from a narrative framework, not only as a dark, formless stage in a personal story.

A focus on the present moment as an emergent site infused with heterogenous lines of force can shift the way one might think of illness and wider biopolitics. Anthropologist S. Lochlann Jain, for example, meets cancer prognosis by refusing a survivorship narrative telos, embracing an alternative ‘elegaic politics’ which moves beyond personal illness story to consider the environmental, sociopolitical and iatrogenic activities which contribute to the increase of breast cancer in the West.^75^ Recent work seeks to combine a respect for the phenomenology of individual experience with a genealogical recognition of the subject as socially constructed.^35 46^ An episodic approach to illness representation and criticism meshes well with these dual goals, in that it may help resist subordinating discrete experiences or sociocultural context to a framing narrative of personal agency. Loosening traditional narrative telos can be part of a more ambiguous positioning of a ‘self’ as dependent on a range of other forces, both human and otherwise.^76 77^ In a similar vein, Willis, Waddington and Marsden, scholars of literature, culture and medical history, have called for an ‘aesthetic epidemiology’ that approaches texts in search not of plot but ‘episodes—independent aesthetic moments given life in language’, rich in intertextual and historical connections.^7^ In this article, I suggest that rather than approach moments in illness narration as either an individual failure to reach self-authorship, or as a temporary stage in an individual journey towards a coherent self and voice, we could approach these scenes as moments within social contexts, dramatising (for example) the structural exclusions afflicting those enduring this condition, the institutional contexts that find their experience unintelligible, and the economic pressures that render many people in chronic pain profoundly precarious.

Representations of chronic pain certainly can lament the experience of being trapped in a present torn from a coherent narrative of past and imagined future, as exemplified in the earlier quotations from Frank, Charon and Nadelhaft. However, I suggest that such texts may also locate their temporal horror differently, in ways that a narrative-focused criticism may conceal rather than help to understand. I am interested in the shifting lines of force (social, emotional and somatic) that shape the affective experience of these moments. To expand our vocabulary for this position, I offer a term for a particular affective experience of the present amid repeated marginalisation: the *temporality of thwarted connection*. This term seeks to convey the experience of a present in which one reaches for connection *—*for diagnosis, medical care, emotional support or companionship amid acute suffering*—*while aware of the (justified) anticipation of imminent failure and future pain, the recollection of past failures and past pain, acute self-awareness of one’s present performativity in the clinical encounter, and one’s ongoing somatic and emotional distress. Diagnosis is often necessary for doctors to offer further care, health insurance companies to fund it, and employers and loved ones to furnish support; as Alison Kafer describes, diagnosis may require ‘shuttling between specialists… repeated refusal of care and services, the constant denial of one’s experiences, the slow exacerbation of one’s symptoms, the years without recognition or diagnosis, the waiting’.^44 78^ As the ensuing literary case study dramatises, attempts to have one’s pain experience validated stand a high risk of failure due to the vulnerability of chronic pain patients as liminal figures who breach diagnostic and social boundaries. Rather than assess this text in terms of its narrative coherence or drama, I want to explore how it depicts particular tormented moments before they solidify into stable ‘meaning’, conveying the complex temporality of an agonised present.

### Lous Heshusius, *Inside Chronic Pain*

Lous Heshusius, the author of *Inside Chronic Pain: An Intimate and Critical Account*, has suffered profoundly from chronic pain for many years. Her pain began after a near-fatal automobile accident in which she sustained significant neck trauma, and her suffering rapidly became so unbearable that she could barely function; at its worst, even moving or speaking became impossible. As a sociologist, she drew on her research skills to explore the biomedical models of pain and the social suffering which can attend pain within her American context. Her book is a blend of academic analysis and autobiography.

Early in a first reading, Heshusius’s text may seem likely to exemplify narrative expectations enshrined within both medical humanities and popular survivorship discourse. She is explicit that she wants a restitution story and describes her efforts to seek one; further, she keeps a diary for years and finds that ‘the ordering process demanded by language… kept me from falling off the edge of life’.^79^ It may seem appropriate, then, to approach Heshusius’s work through the ideas of self-authorship so influential in narrative medicine. Yet at the same time, her writing undercuts those frameworks. She resists the framework of ‘quest’ narrative, not ‘honouring’ her pain ‘for the sense of purpose that can be discovered in it’, in Frank’s phrase, and warning that ‘the world also needs to witness the stories of pain that go on with no end in sight…. Those that end in despair, in death…. The entire range of pain stories needs to be acknowledged to encourage political and social progress’.^79^ In this telling, an advantage of unhappy narrative is its potential activist power. However, more than simply calling to diversify pain stories, Heshusius’s text undermines linear and teleogenic narration in multiple ways. Most significantly for this discussion, she powerfully conveys the affective strain of *moments* of suffering, demanding attention to particular episodes of agony without taming these within a story of personal transformation or enrichment.

The complex temporal structures of Heshusius’s text dramatise how chronic pain is, etymologically, a pain of time, of tortured temporality as well as body. While few narrations are strictly chronological, since all feature flashback and prolepsis, in this case the structure itself is explicitly thematic, focused around key elements of her experience (pain medicine, healthcare practitioners, pharmacology, social relationship, etc), and each chapter features personal story interwoven with sociological academic study. Within each chapter, the temporality of the discussion is highly fluid, interweaving early and late experiences, and including dreams in their vivid temporal jumble. Heshusius describes a Kafka-esque search for diagnosis and effective treatment, ‘feeling as if I were on a treadmill, going from doctor to doctor, feeling no one spent enough time with me to understand my problems. Twice I went to the wrong hospital, as hospitals and clinics blurred in my mind. The buildings all looked alike. The doctors all seemed to do the same thing’; ‘their responses have been, more often than not, contradictory. Maddening at times. I have walked out of their offices in utter confusion. What to believe now?’^79^ Yet the connection Heshusius seeks is not merely the diagnostic event. She poignantly yearns for tenderness, for practitioners to be ‘kind’, and describes how appointment time constraints thwart the dialogue so necessary for healing and support; she experiences tight time constraints as violent, speaking of the ‘Medical Slaying of Minutes’.^79^ She suffers profoundly from both physical pain and the attendant isolation and sense of delegitimation, and she repeatedly describes craving death. Heshusius’s work can certainly be approached as dramatising the way that illness experience can destroy one’s sense of a coherent narrative self. However, we can complement such an interpretation with an *episodic* reading, alert to the present as not yet an event but rather a suspended impasse, a waiting and reaching, within a very particular hostile social and medical milieu. I will briefly consider two scenes.

Heshusius describes repeated failed attempts to have her pain acknowledged. On one occasion she tries to explain the experience:

I try to speak to doctors about the severity of my pain. My words float strangely in the air. As I pronounce them, I myself become a spectator. As soon as I begin to speak, I am no longer there. Someone else is speaking these words. Someone who has not suffered the pain, for it is much worse than she says. How can she say so little? … In the meantime, I am watching the doctor. Trying to see how he reacts. Did he get it? Should she be more dramatic? More detailed? But how? How can she, how can I, express this prelanguage torment?^79^

Heshusius dramatises the temporality of thwarted connection in her descriptions of repeated delegitimation. As Lara Birk notes, ‘patients must perform their pain. To be credible, the sufferer must act out her pain…. Yet it is the inescapably performative nature of the behavior that actually kills the very credibility one seeks to guarantee’.^80 81^ Aware of past failure, dreading an imminent repeat, scrutinising her present ‘performance’ and the practitioner’s response, Heshusius brings her experiences together under a sign of erasure, of annihilation. Her pronouns are unstable: initially she is I, until her words enter the communicative space to be heard by another. The profound vulnerability of that position is experienced as violent erasure*—*‘I am no longer there’*—*and she becomes detached from and even critical of the speaking woman (‘she’). Yet there *is* still an I*—*a rightly apprehensive subject, watching the doctor and trying to gauge if the performance is adequate. Her closing lamentation is poignant. How to communicate this lived reality in the face of repeated disbelief, non-comprehension and despair? Here stable meaning is elusive and recognition is lacking, but Heshusisus’s prose in this fragment conveys her *labour* amid the suspended moment of chaos, the burden to communicate and to endure this moment fraught with intersecting lines of force affective, social and cognitive.

A second fragment also conveys the affective complexity of the emergent present at a particular moment in her chronic pain experience:

During my worst four years, every day resembled dying. Even now, I often feel strangely close to death. …because this life in pain has asked of me to part from nearly all that I thought constituted my life. Death as *afscheid nemen.* I have to say this in Dutch, my mother tongue, to capture what I mean. To say goodbye, to take leave in the deep sense of parting *—* parting for a very long time, perhaps for ever, from people, from places, from activities that are very dear. This parting as an ongoing process often renders an aloneness that feels total and so numbing that it brings on more despair and more pain. Isolation, and the taboo that slowly develops about talking about one’s pain to others, intensifies inner turmoil and intensifies the pain itself.^79^

Here, too, Heshusius exemplifies the temporality of thwarted connection*—*an agonised present informed by past failures to connect and apprehension of ongoing alienation. As before, she brings the complex temporality of the moment together under a sign of a self erased, excluded, gone. She describes inhabiting the present as an ongoing farewell*—*her daily life is ‘*afscheid nemen*’. This trope conveys profound temporal complexity: a leave-taking acknowledges an imminent event of separation, a future without that person. Yet Heshusius uses this metaphor to describe not an imminent event but an ongoing state*—*an agonised leave-taking, prolonged and unfinished, for a person whose pain is taboo.

Heshusius’s book is necessarily harrowing and relentless, but it does describe moments of relief. What is unexpected is that several of these consolations are antithetical to the satisfactions of narrative medicine. First, she experiences a moment of mindful awareness that overflows with solace. As Mark Sullivan and David Zucker explain, mindfulness requires resisting turning experience into narrative. Heshusius finds this state profoundly beneficial, describing a ‘disappearing me’, ‘surrendering these things called “I” and “pain”’.^79^ Furthermore, writing also yields benefits to Heshusius that are not best read as functions of a ‘coherent’ narrative self. It offers a relief to the degree that it helps her feel heard and witnessed *in the moment*. Heshusius finds that her diary ‘gave me both an intimate “other” to go to… My scribbles are there. They cannot run away, as have friends and colleagues, as well as many doctors. They are waiting for me’.^79^ Her text is a plea for witness of moments of suffering. Finally, Heshusius describes how attending to the fragmentary moments presented through her diaries can produce something different from the usual narrative arc of a personal journey:

as my story takes shape, there are many moments of despair, bewilderment and grief and many moments of feeling abandoned by doctors and friends alike. Often these moments appear as they did in my journals in all their sharpness. I ask the reader to bear with me, as I slowly learn to place these moments in the larger contexts of cultural and human complexities and suffering of all kinds.^79^

Here Heshusius positions her diary fragments not within a coherent story of self, but rather within a wider cultural context. Fittingly, then, she ends her book by discussing current activist efforts to raise awareness and transform funding for chronic pain research.

If we read Heshusius’s text in search of an effort to restore a narrative sense of self, what we find is a failure, a refusal to ‘honour’ illness ‘for the sense of purpose that can be discovered in it’.^42^ However, if we read it episodically, open to the affective weight of the moments she describes, then the emphasis shifts to the tortured temporality of repeated efforts of connection, the profound need for a transformed social and medical response to those living with chronic pain, and the affective complexity of moments of illness experience before narrative can emerge.

In considering reader position, my article overlaps to some extent with Claire McKechnie’s defence of narrative as central to any reader’s interpretative work.^5^ McKechnie approaches narrative as describing any act of successful communication or ‘transmission of an idea’, and she calls us to recognise the narrativity of multiple media. McKechnie also argues that any reader must inevitably respond to a text using a narrative framework. To illustrate her point, McKechnie offers a sensitive analysis of an excerpt from Dennis Potter’s reflections on the way his awareness of dying has transformed his perception of the natural world, in which he describes noticing the splendour of apple blossom. McKechnie argues that the work of reading and understanding these words by Potter inevitably requires a reader ‘ordering information’ into ‘story’: ‘It is only through narrative that we gain access to Potter’s world and get a sense of what he is experiencing’.^5^

I share McKechnie’s eagerness to see a broader understanding of narrativity as applicable to a multitude of media; and if narrative is taken broadly to mean any act of meaning-making, then I agree that any reader makes a narrative*—* although I suspect that defining all acts of meaning-making as narrative might diminish a useful specificity in the term. Either way, however, I want to notice and unsettle the assumption that the act of reading must incur an orientation towards teleogenesis, an assumption about how a textual passage is oriented towards a particular unfurling in imagined future time. The narrative McKechnie gleans from Potter’s words is highly teleogenic, reading his description of blossom as ‘captur[ing] the transitory and temporary nature of life and the inevitability of death and decay’. I agree this is a valid story that a reader could feel rise within themselves as they read Potter’s words. However, what I argue in this present article is that such a teleogenic narrative framing by the reader is not the *only* viable response. Indeed, while McKechnie’s reading finds his description of the blossom to be about how life is ‘beautiful, but it is fleeting’, Potter’s words in the extract she cites do not describe the blossom as ephemeral or include any bittersweet contemplation of the way they will fade. Rather, his focus is on the blossom as extraordinarily vivid, ‘the whitest, frothiest blossom that there ever could be, and I can see it’.^5^ Without dismissing McKechnie’s thoughtful reading, I suggest it could be complemented by an episodic reading, dwelling with the scene as *a moment in itself*, not only implying future decline, not only about a temporal trajectory, but about something instant, present, embodied and *now*. Potter himself invites exactly such a response, exclaiming, ‘The fact is, if you see the present tense, boy do you see it!’^5^ In this fragment, Potter does not subordinate his enhanced awareness to his imminent death. To say it another way, to read episodically is to recognise that the meaning of a scene may not stem only from its sequel.

## Conclusion: staying with the trouble

Describing the need for new ethical modes of being on human-damaged earth, Donna Haraway calls for ‘staying with the trouble’, to develop a capacity to remain with the distress and tumult, choosing to focus on the present.^82^ Haraway formulates this concept within her cultural studies work on ecological devastation, but I find her phrase invaluable in this different context. I suggest the phrase can also capture the challenge and promise of an episodic, moment-focused reading. In such a reading, one does not seek to move too quickly to discerning a narrative framework, wary of the way such framing can leach the painful affective complexity from a representation of an emerging present.

It is difficult to hear suffering without imposing a narrative framework.^83–85^ Indeed, narrative medicine has shown that narrative typologies can be invaluable in the way they help hearers/readers to better notice aspects of patient experience. While I greatly respect this approach, I suggest that it can fruitfully be complemented by an episodic focus, letting disturbing moments stand alone before taming them within a narrative of progress, personal meaning or other telos and consolation. As a parallel to ‘flash writing’, here I am urging what might be called ‘flash reading’, a willingness to surrender*—* even if briefly*—*to the instant of the textual encounter, to the passage, the excerpt, the troubling episode, and to let that extract sit with you, remain with you, haunt you, without closing it off within a narrative arc. I do not suggest this is a novel approach*—*this kind of episodic reading has always been part of a reader’s repertoire of response. Rather, I am seeking to name it and to bring it more consciously into the strategies of our analytic discourses, specifically in the hope of disrupting certain axioms of illness narrative study, resisting inadvertent hierarchalisation of illness experience, and crucially, feeling towards an articulation of the way illness experience and narrations can be seen as an emergent present infused with lines of force.

Ann Jurecic, a scholar of literature and life writing, warns that literary criticism of pain has often failed to consider the question of ethical response. Following Scarry’s formulation of pain as annihilating the world, critics have tended to read representations of pain for the way they convey the experience as incommunicable, failing to recognise that what these narrations often seek is not someone to understand the specific nature of the pain, but rather to acknowledge the reality of the suffering.^86^ Sharing Jurecic’s concern, I suggest reading episodically can also lead to a different sense of the affective response of a reader/auditor: not to respond to suffering with ‘you are so brave’, or even with ‘your pain is a mystery’, but with ‘I believe you suffer and I stand beside you’. This kind of reading/hearing stance may also have value in the context of encounters between healthcare practitioners and patients. Catherine Belling has suggested that lyric may be more helpful than narrative for describing the process of reflective practitioners, capturing the need to ‘pause the momentum of plot and to focus down, observe closely and question deeply’.^1^ In a similar vein, I would suggest that alongside narrative competence, practitioners need to be aware of how listening for narrative also has risks. Rather than foregrounding the coherence of some illness representations, we can recognise how value may inhere in the rupture and the breach. We can heed these traces of embodied suffering before they solidify into story.
